# Navigating the landscape of non-health administrative data in Scotland: A researcher’s narrative

**DOI:** 10.12688/wellcomeopenres.15336.2

**Published:** 2019-10-21

**Authors:** Matthew H. Iveson, Ian J. Deary

**Affiliations:** 1 Centre for Cognitive Ageing and Cognitive Epidemiology, The University of Edinburgh, Edinburgh, UK; 2 Administrative Data Research Centre Scotland, Edinburgh, UK; 3 Centre for Clinical Brain Sciences, The University of Edinburgh, Edinburgh, UK

**Keywords:** Administrative data, Big data, Data linkage, Narrative, Social epidemiology

## Abstract

**Background:** There is growing interest in using routinely collected administrative data for research purposes. Following the success of research using routinely collected healthcare data, attention has turned to leveraging routinely-collected non-health data derived from systems providing other services to the population (e.g., education, social security) to conduct research on important social problems. In Scotland, specialised organisations have been set up to support researchers in their pursuit of using and linking administrative data. The landscape of administrative data in Scotland, however, is complex and changeable, and is often difficult for researchers to navigate.

**Purpose:** This paper provides a researcher’s narrative of the steps required to gain the various approvals necessary to access and link non-health administrative data for research in social and cognitive epidemiology.

**Findings:** This paper highlights the problems, particularly regarding the length and complexity of the process, which researchers typically face, and which result in a challenging research environment. The causes of these problems are discussed, as are potential solutions.

**Conclusions:** Whereas the potential of non-health administrative data is great, more work and investment are needed on the part of all those concerned – from researchers to data controllers – in order to realise this potential.

## Introduction

The rise of big data represents a revolutionary opportunity for both researchers and policy makers. This opportunity has been perhaps best recognised by Scandinavian countries (Sweden, Norway and Denmark), in which national databases – including healthcare and conscription data – have been linked together using unique personal identification numbers, allowing for large and powerful research studies ^1^. These studies have significantly improved our understanding of issues such as cancer ^2^, mental health conditions ^3^, pre-term birth ^4^, cognitive ageing ^5^, socioeconomic inequality ^6^, etc. In Scotland, previous work has already leveraged routinely collected health-related administrative data, such as that from the National Health Service, to address questions regarding how morbidity and mortality relate to people’s social background and psychological differences ^7– 10^. Health data research has benefitted from increasing investment (from both governments and research councils), and from several high-profile public promotions (e.g., the ‘data saves lives’ campaign). In the last decade, researchers have extended their sights to routinely collected administrative data, such as that from the Scottish Government, as a largely untapped resource with similar potential for impact and societal benefit. These requests have been facilitated by purpose-built organisations such as the Administrative Data Research Centre Scotland (funded by the ESRC). The role of these new organisations is to support researchers and to negotiate access to both health and non-health administrative data on their behalf. Furthermore, many of the organisations controlling non-health administrative data have begun to develop and implement processes for dealing with data requests. In contrast to earlier efforts, then, data access and linkage for research purposes should be easier and faster. However, despite the promise of non-health administrative data, the road to obtaining data is not always smooth.

**Figure 1.**
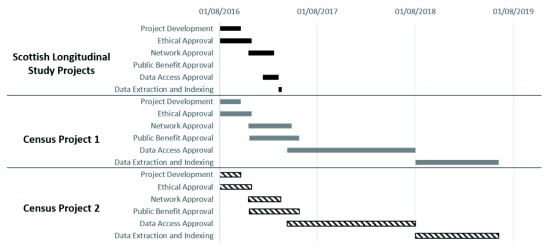
Gantt chart demonstrating the time taken to complete key stages for each project.

Below we give a researcher’s perspective on the journey through the landscape of administrative data in Scotland. The narrative describes and comments on a project devised to link the Scottish Mental Survey 1947 cohort (SMS1947) ^11^ to routinely collected health and non-health administrative data, including the Scottish Census. This follows on from and extends similar efforts to link the same cohort to routinely-collected health administrative data, carried out before major changes in the Scottish landscape of big data ^8– 10, 12^. While previous efforts have used linked SMS1947 and health data to investigate life-course determinants of cause-specific mortality ^9^, the current project sought to extend this linkage to non-health administrative datasets and use them to examine health and social care outcomes. The present account, then, is partly an update, now that data linkage organisations and processes in general are more mature, and also a major extension, given that access to non-health administrative data is a relatively recent development. The post-doctoral researcher employed as part of this project was in post for 26 months, from 1 ^st^ August 2016 to 1 ^st^ October 2018. We describe the process involved in acquiring and linking data for four specific studies (see Figure 1) – two involving data from the Scottish Census and two involving data from the Scottish Longitudinal Study (SLS). The SLS is a standing resource containing pre-linked records for a 5.3% sample of the Scottish population, covering primarily census data, education data and hospitalisation data (see Box 1). As the SLS offers pre-linked data, data access approval and data extraction and indexing are much faster than non-SLS projects ( Figure 1). The two SLS studies were conceived as interim studies to be conducted while completing the approvals process for the two census studies, and are included for comparison. The two census projects differed slightly in the specific datasets to be linked, though the cohort to be linked was the same. The organisations involved, their role, and they type of data they hold are summarised in Table 1. Note that we do not claim to be experts in the legalities and technicalities that motivate the information governance and data access processes described below; that is an unrealistic expectation to place on researcher. Instead, we describe the process as it is faced by researchers and reflect on the challenges that arise along the way. We provide this narrative in the hopes that it will inform researchers who are considering working with non-health administrative data, and that it will help to critically assess and improve current data governance and access policies.

Box 1. **Challenges to using the safe settings.**

Given the time required to arrange the linkage of the full Scottish Mental Survey 1947 cohort, we pursued projects using a sample of the cohort which had already been linked within the Scottish Longitudinal Study (SLS). As the SLS is a standing database of linked health and non-health administrative data, covering 5.3% of the Scottish population selected from 20 dates of birth, we reasoned that these projects would be quicker to obtain and analyse data. Note that the approval and access process is very different for SLS data than for data which has not been previously linked; procedures have been pre-agreed between data controllers and there is only a single point of contact. The SLS also provide research support functions to all users. Researchers using SLS data are required to do so in a ‘safe setting’ – a secure, monitored environment within Ladywell House (Edinburgh, UK). Data can only be viewed from this safe setting, and all analyses must be done on-site and later checked for potential statistical disclosure before being removed from the safe setting. We first visited the safe setting on 21 ^st^ March 2017. Over the course of using the safe setting (442 days; from 21 ^st^ March 2017 to 6 ^th^ June 2018) we ran into several issues which lengthened the planned projects. Below we summarise these issues. Whereas some are specific to those projects using the Scottish Mental Survey 1947 cohort, the majority of the issues listed below reflect the type of trials faced by all users of the SLS safe setting, and of access-controlled data sites more generally (e.g., the ADRC-S safe havens).

**Approval:**

- Approval required for the project itself (1 form) and for the researchers themselves (2 forms)
- Additional aim required formal approval from SLS panel (submitted 11/04/2017). Approval was not communicated to the researchers until later (04/05/2017)

**Availability:**

- 3 delays in attendance due to SLS staff shortage/training

**Changes in policy:**

- Booking space in the safe setting changed to require 2 days’ notice (12/10/2017)
- Intermediate output minimum cell count changed from 5 to 10 (02/11/2017)
- Disclosure control timelines changed from 5 working days for intermediate output and 15 working days for final output to 10 working days and 20 working days (29/03/2018)

**Analyses:**

- Initial dataset was missing a requested variable (21/03/2017). A new dataset was extracted (23/03/2017), including the missing variable, but was not made available until later (02/05/2017) due to staff shortage. A second missing variable was identified (25/05/2017) and was later added (12/06/2017).
- Analyses was conducted in R Studio using specialist packages ^13^. These packages were not pre-installed on the safe setting machines, and needed to be requested (7 forms).
- Although the packages were installed, it emerged that their dependencies were not. These dependencies had to be subsequently requested (2 forms).
- The installed version of R Studio was not compatible with some of the installed packages, and so a newer version had to be requested (1 form)
- Some analyses were not included in intermediate statistical disclosure controlled output (18/05/2017)
- 4 intermediate outputs were redacted due to concerns over statistical disclosure. Concerns were raised particularly regarding the inclusion of Ns, despite these adhering to the disclosure control guidelines (all cells greater than 10, or censored accordingly).
- Concerns were also raised due to the cohort used – multiple projects working on the same cohort can produce tables which, when combined, are said to risk residual disclosure. Giving current researchers particular outputs may mean that future researchers are prevented from producing other outputs.

**Table 1.** Organisations, their role, and number of forms required. Merged cells indicate shared involvement.

Organisation	Role (Type of data held)	Forms submitted	Amendments submitted
Administrative Data Research Centre - Scotland	Research Support	0	0
ACCORD	Clinical Sponsor	1	0
NHS Research Ethics Committee	Ethical Approval		0
University of Edinburgh Legal Services	Institutional Guarantor	1	0
Administrative Data Research Network	Network Approval and Resources	5	0
Public Benefit and Privacy Panel	Public Benefit and Privacy Approval	2	5
NHS Information Services Division	Data Controller (Health data)	0	0
Electronic Data Research and Innovation Service	Research Coordinator for NHS ISD	0	0
National Records of Scotland	Data Controller (Births and Deaths data)/Trusted Third Party	4	0
Scottish Government	Data Controller (Census data)		0
Scottish Longitudinal Study	Data Resource (Pre-linked administrative data)	2	1

### Stage 1: Ethical approval (prep time: 3.5 months; processing time: 0.5 months)

The conception of the project began on 1 ^st^ August 2016 (see Figure 1). Because pseudo-anonymised health data linkage had been achieved for the SMS1947 cohort as part of a previous project ^8– 10^, the first step was to determine whether the ethics and permissions obtained previously could be extended to cover the proposed project. Importantly, previous projects obtained specialised ethical approval due to the unconsented use and linkage of health administrative data. After several weeks of meetings and emails with colleagues in the Administrative Data Research Centre – Scotland (ADRC-S), NHS Information Services Division (NHS ISD), ACCORD (the clinical sponsor), and the NHS Research Ethics Committee (NHS REC), it was determined that new specialised approvals would need to be sought. This period reflected the relative unfamiliarity of some of these organisations with data linkage projects, and the conflicting interpretations of data linkage procedures. The researchers submitted an ethics application covering the new project to the NHS REC for initial review on 21 ^st^ October 2016, and for final review on 18 ^th^ November 2016. The ethics application was formally approved 11 days later, on 29 ^th^ November 2016.

### Stage 2: Network approval (prep time: 3 months; processing time: 0.5 months)

The next stage of the process was to obtain approval from the Administrative Data Research Network (ADRN) in order to be able to access their support and infrastructure. This involved the preparation of a second set of forms – one for each of the two census studies and one for the SLS studies within the project. We began preparing these forms on 17 ^th^ November 2016 and submitted iterative versions to the ADRC-S for preliminary feedback on the 20 ^th^ December 2016 and on 12 ^th^ January 2017. Final versions of the forms were submitted to the ADRN in turn, from March through April of 2017. Approval for each study was obtained roughly 2 weeks after submission, with the final study being approved on 27 ^th^ April 2017. Whereas these forms were to gain ADRN approval for the proposed studies, a third set of forms was required to gain ADRN approval for the researchers involved. This approval requires researchers to detail their research experience, to detail any previous incidences of data misuse, and to agree to abide by the ADRN’s terms of use. Note that these forms were in addition to the research governance training courses already undertaken as preparation for the project. These forms were started on 6 ^th^ February 2017. These ‘approved researcher’ forms had to be approved by the institutional guarantor, ensuring that the research institution supports the researchers and adopts the responsibility for any misconduct, prior to being submitted to the ADRN proper. Institutional approval was granted on 10 ^th^ February 2017; final ADRN approval was granted that same day. Note that this stage is no longer required since the conclusion of the ADRN, reflecting changes to the approvals process since the projects were undertaken.

### Stage 3: Public benefit and privacy panel approval (prep time: 4.5 months; processing time: 1.5 months)

To ensure compliance with data protection law, it is necessary to demonstrate both a legal gateway by which data can be provided by data controllers (see *Stage 4* below) and a public benefit resulting from the research. Stage 3 of the linkage process dealt with obtaining permissions for data linkage and use from the Public Benefit and Privacy Panel (PBPP), whose role it is to weigh-up the potential benefits arising from proposed research projects against the risk of breaches in privacy. The PBPP provides a single-point of application for permissions regarding health administrative data, where previously approval was required from the Caldicott Guardian of each health board involved ^12^. Notably, this process was only required for the two census studies, as assessment of the public benefit and privacy of the SLS studies was combined with the data access approval process (see *Stage 4*). This is one of the key ways in which the process required by the SLS projects differed from the two census projects. We began drafting a single PBPP application form on 26 ^th^ August 2016 with a view to simply amending the existing permissions for linkage and use established by previous work with the SMS1947 cohort ^12^. However, it became apparent during the ethics process (see *Stage 1*) that the new project necessitated new approvals, and so two separate PBPP forms were drafted, one for each of the census studies. Note that two forms were required as the two census project addressed different research questions, despite the similarity in the datasets to be linked. We began these drafts on 21 ^st^ November 2016. Initial drafts were submitted to the ADRC-S for feedback on 15 ^th^ February 2017. Following extensive feedback from the ADRC-S support staff (each form was revised four times, from 23 ^rd^ February to 9 ^th^ April 2017), these two forms were submitted to the PBPP on 10 ^th^ and 12 ^th^ April 2017. Conditional approval was granted on 23 ^rd^ May 2017, and the required amendments were re-submitted on 25 ^th^ May 2017.

### Stage 4: Data access approval (prep time: 2 months; processing time: 14 months)

After being granted ethical, network, and PBPP approval, the last approval to be obtained is that of the data controllers. Given that, to get to this stage, our studies had already been deemed ethical, feasible, legal, in the public interest, and reasonably secure, approval from the data controllers themselves might be considered to have been relatively trivial. In the case of some organisations this was, indeed, the case. The SLS studies were approved within 2 months of beginning the application process (see Box 1 and Figure 1). Again, this is thanks to the pre-linked nature of the SLS data and the unified approvals process. For the two census studies, NHS ISD – the organisation holding the majority of the health administrative data required by the studies – required no further approvals beyond those already obtained. The electronic Data Research and Innovation Service (eDRIS), acting as research coordinator for NHS ISD with regard to the requested health data, requested only proof of information governance training (i.e., certificates of completion). Approval from the individual acting as data controller for the Scottish Mental Survey 1947 was obtained on the same day as the relevant form was submitted – 13 ^th^ March 2017.

Access to non-health administrative data, however, proved to be much more complicated. Four additional forms – a Privacy Impact Assessment and a Data Access application for each of the two census studies – were required by NRS and Scottish Government. Note that Privacy Impact Assessments have since been replaced by Data Protection Impact Assessments, and are now required for all new data linkage projects in the UK. We began drafting these forms on 11 ^th^ April 2017 and sent them to the ADRC-S for initial review on 27 ^th^ April 2017. After making changes according to the advice of ADRC-S support staff, finalised forms were sent to the ADRC-S on 16 ^th^ June 2017. These forms, however, could not be submitted directly to the organisations and, instead, entered a queue. At the time, NRS and Scottish Government would only accept small ‘batches’ of around five projects at a time in order to avoid overloading their capacity; all projects within a batch would need to be processed and approved (or not) before the next batch would be accepted. As such, the ADRC-S retained a queue of batches ready for submission. Our two studies were part of the second batch, and so had to wait for the first batch to be examined and cleared before being submitted, let alone considered. The first batch was cleared on 7 ^th^ July 2017, and the Data Access and Privacy Impact Assessment forms were submitted formally on 19 ^th^ September 2017. On submission, these forms were distributed to the NRS Privacy Group, the Scottish Government Statistics PBPP and the Scottish Government lawyer for simultaneous assessment. On 4 ^th^ December 2017 the presiding Scottish Government lawyer left the post, leading to a delay until the post could be filled. A new lawyer came into post in January, although this necessitated a reassessment of the forms by the new lawyer. While being considered by the Scottish Government legal team, Scottish Government Statistics PBPP approved the two census studies on 22 ^nd^ January 2018. On 2 ^nd^ March 2018, it became apparent that the new Scottish Government lawyer was unwilling to accept the legal gateway identified by census studies (Section 5 of the Census Act (Scotland)) or to approve the second batch based on the precedent of the first. Further investigation would be required to identify a new, more appropriate legal gateway for sharing census data. At this point, it was unclear how much time would be required for this investigation and how much of a delay would result. Due to the risk that census data would become available beyond the lifespan of the project, we decided to continue with the linkage between the other data sources for the two census studies. This necessitated an amendment to already-submitted PBPP forms (see Stage 2), which was submitted on 29 ^th^ March 2018 and was approved on 3 ^rd^ April 2018. However, a new legal gateway (Section 4 of the Census Act (Scotland)) was identified on 3 ^rd^ April 2018, and the Scottish Government lawyer gave their approval for the two census projects on 17 ^th^ April 2018.

The census projects were then passed to the Scottish Census Privacy Working Group, who review the privacy and security arrangements of studies. On 23 ^rd^ May 2018, the Scottish Census Privacy Working Group asked for a revision of the intended census data retention period from 5 years (as per the eDRIS and National Safe Haven policies) to 2 years. An amendment to this effect was submitted on 25 ^th^ May 2018, and access approval was gained in August 2018.

### Stage 5: Data extraction and indexing (processing time: 10+ months)

After approval, data needs to be extracted and indexed before it is made available to the researcher. This process largely occurs ‘behind the scenes’, and is coordinated by the Trusted Third Party to help ensure privacy and minimise the transfer of personal data. Indexing – the process of assigning a random, unidentifiable index to each individual – was completed in May 2019, several months after the end of project funding, and only for one of the census projects. Indexing delays have partly resulted from demand and staffing issues within the Trusted Third Party team (NRS Indexing). Although these indexes are now with data controllers for use in data extraction, no data can be transferred to the safe haven infrastructure until data sharing agreements are signed. These the legal contracts, which lay out the responsibilities of the organisations and researchers, are still being drafted and agreed between data controllers. As a result, the prospect of analysing data is still some way off. Meanwhile, access to SLS data was provided around 2 weeks after data access had been approved, and analysis was largely completed around 6 months after the data was made accessible.

## Issues

### Timing

One of the most important issues highlighted by the above narrative is the time taken to achieve non-health administrative data linkage (from 1 ^st^ August 2016 to 7 ^th^ June 2019 currently; see Figure 1). To date, the above project has taken 34 months, even before gaining access to the requested data. Stage 4, data access approval, has by far taken the most time, although it does not mark the end of the administrative process. The exception has been obtaining SLS data. As a standing database, the SLS has the advantage of well-established protocols and there being a single point of application. However, researchers may still face challenges when gaining access to and using SLS data (see Box 1). Furthermore, the restricted scope and relatively small sample size of the SLS may not be suitable for all researchers.

Previous efforts to obtain and link routinely-collected Scottish data for research purposes has been lengthy and complicated (e.g., 538 days) ^12^. More recent changes in the Scottish data landscape, such as the Public Benefit and Privacy Panel, which replaces individual Caldicott Guardian approvals, should have improved the experience for researchers. This has generally been the case in regard to health administrative data, though data access is still prone to delays. For non-health data, whereas the number of forms to be submitted and organisations to be contacted has been reduced, the amount of time taken to obtain linked data has remained largely unchanged. Admittedly, the linkage project described here was much more ambitious than previous projects using the SMS1947 dataset and involved the linkage of more datasets from more data controllers. Notably, the current timescales are problematic for those conducting the research, particularly in academia where funding is time-limited. For the above project, data was not obtained before the end of the funding period and the post-doctoral researcher’s contract. Taking the presented timescales as representative, the current situation essentially prohibits researchers, particularly early-stage researchers, from conducting projects involving linked non-health administrative data unless permissions are sought well in advance. For example, a full-time PhD student would have been required to submit their thesis within the time taken to obtain linked non-health administrative data.

Note that timing also affects those organisations set up to aid researchers in acquiring data. For example, the ADRC-S was funded for defined periods (1 ^st^ October 2013 to 1 ^st^ October 2018; by the ESRC), and was refunded but fundamentally re-specified within the lifetime of the described project (1 ^st^ October 2018). At the same time, the funding for the ADRN was not renewed. These organisations were themselves preceded in Scotland by the Administrative Data Liaison Service and have since been superseded by the Administrative Data Research Partnership and the Scottish Centre for Administrative Data Research (SCADR). These organisations are judged on their success in obtaining new sources of administrative data, and on the number of research projects which are completed with their support. The changes to such organisations therefore reflects the challenges they face in producing results within their periods of funding, given the time taken to obtain data ^14^. An unintended consequence of these changes has been the loss of much of the documentation which researchers use to learn about available datasets and necessary processes and which enable reproducible research ^15^. In its lifetime, the ADRC-S supported over 70 projects, over 10 of which have now obtained data (linking over 25 distinct datasets) with over 40 projects still being sought under the new SCADR structure.

### Process

The long timescales, particularly in the approval of data access requests, in part result from the relative infancy of the non-health administrative data landscape in Scotland. Whereas health administrative data controllers have developed clearer and more streamlined processes, non-health administrative data controllers are not yet at this stage. For those seeking only health administrative data, ethics, PBPP, indexing and extraction are required, though this process can still be lengthy ^12^. However, where PBPP acts as a single point of health administrative data permissions, those seeking non-health administrative data must negotiate with and complete the governance procedures of all concerned data controllers, resulting in a complex and often unclear process.

The project described here has, to date, necessitated the submission of some 21 forms, including amendments ( Table 1). For the most part, non-health administrative data controllers have been reflexively developing processes as data requests are submitted, and these processes have been prone to significant change. For example, the NRS and Scottish Government developed a process for dealing with census data requests in response to the first project submitted to them, a project examining end-of-life care (Schneider and Atherton, *In Preparation*) initially submitted in December 2015. As part of this new process, NRS and Scottish Government identified a legal gateway (Section 5 of the Census Act (Scotland)) necessary to allow them to share census data to researchers. Subsequent requests for census data followed this process, citing the same legal gateway. However, these projects were not provided with data due to a change in Scottish Government’s legal interpretation regarding the appropriate legal gateway (see above). Such reflexive changes to policy and process by non-health administrative data controllers indicates the uncertainty with which they have taken to data sharing. Similar changes were seen during the development of health administrative data linkage procedures around 2013 ^12^. These changes also reflect a problematic culture within organisations in their perception of risk and public interest. Previous reviews note that data controllers often design processes that disproportionately restrict data sharing in order to account for barriers to data sharing, whether real or perceived ^16^. While it is important to get procedures right, unnecessary complexity and unexpected changes often lead to delays and damages the trust between researchers and data controllers. Trust requires appropriate, and not excessive, governance that can be well-understood by both sides of the process ^17^. Whereas processes will continue to change as non-health administrative data controllers mature, the issue of trust may be partly addressed by their greater engagement early in the life of a research project. For example, data controllers could give conditional guarantees for data at the start of the permissions process, contingent on the research project acquiring ethical, public benefit and privacy approval. While this process would still need to respond to changing procedures, it would give some level of certainty for researchers and would ensure accountability for any subsequent delays or failures to provide data. However, appropriate incentives would be required to encourage such collaboration.

It is worth noting that some degree of complexity is necessary given the sensitivity and scale of the data being requested and the resulting risk to privacy and impact of improper use. The variety of data access procedures described here are in place to protect the privacy of individuals and their data, and it is important that researchers demonstrate their plans for and commitment to minimise any risks. Furthermore, data controllers such as NRS have responsibilities to care for the data under their charge which outweigh their responsibilities to share data for research. We therefore do not suggest that the data access process should be less thorough or strict. Indeed, streamlining the data access process (e.g., uniting all non-health data access approvals) should not come at the cost of an increased risk to data privacy nor at should it damage data controllers’ responsibilities or reputation. However, improvements can be made to make the journey to obtaining data clearer and smoother. Reflecting on access to health administrative data, an early attempt to link SMS1947 records to routinely-collected health records found that the process took almost 2 years and required some 210 documents ^12^. Since this time, the processes for accessing health administrative data have been streamlined through the development of standardised forms and the development of the PBPP as a single point of application for data access approvals in Scotland. While there can still be setbacks in accessing health administrative data – particularly for complex projects with multiple data sources – the overall result has been a faster process with fewer forms, as highlighted in the narrative presented here. Similar developments could benefit access to non-health administrative data as well as access to data in the rest of the UK.

Though necessary, the complexity of the permissions process (time, number of organisations, potential hurdles, etc.) creates a large barrier to entry for researchers. The current landscape necessitates a guide to identify the required points of application, to lay-out the process for each organisation, and to ensure applications are made in the most efficient order. Whereas research support officers in organisations such as the ADRC-S can (and do) help in this regard, this relies overly on the expert knowledge of specific individuals and on the existence (and capacity) of these support organisations. The future of non-health administrative research, then, is fragile: without clear and consistent processes, and without help to guide them between processes, new researchers would doubtless be lost.

### Capacity

The relative infancy of non-health administrative data research is also reflected in the processing capacity of approvals panels and data controllers. Processing applications, indexing records and extracting data all require resources on the part of the organisation, both in terms of staffing and infrastructure. These resources are finite, and many of the organisations struggle to keep up with the rapidly increasing demand for non-health administrative data. Indeed, several organisations within the permissions and indexing process operate ‘queues’ for research projects. Many non-health administrative data controllers are expected to deal with data sharing requests using existing resources and funding, resulting in a reliance on staff who have other responsibilities beyond data sharing or on relatively small teams. In order to resolve the capacity problem and to speed up processing requests it is imperative that organisations commit more and dedicated staff and infrastructure, and that their funding enables these developments. This is not a new problem, and has been highlighted by previous initiatives in non-health administrative data ^14^. Furthermore, this problem has already been recognised in regard to health data, and the capacity of health administrative data controllers is beginning to increase (e.g., “The research strategy for health and healthcare”, Scottish Government, 2009); a similar effort needs to be made regarding non-health administrative data to help data controllers to deliver on their promises. Although recent investment has been made by the UK and Scottish governments in the form of the Administrative Data Partnership, this needs to be sufficiently targeted towards capacity-building and staffing to maximise the impact on data access.

### A global perspective

Although the narrative presented describes the process of acquiring administrative data in Scotland, many of the experiences and challenges are common to other countries seeing an increase in administrative data research. Although Scotland is at the forefront in terms of the variety of health and non-health administrative data available to researchers, the process of obtaining data is largely the same in other countries. In England, for example, ethical and public benefit approvals are still needed before administrative data access requests will be considered and data extracted, although such approvals are sometimes regional and the Office for National Statistics and NHS Digital play a role in data coordination and linkage. The exception to this is perhaps in Scandinavian countries such as Sweden, in which health and non-health administrative data been utilised by researchers for much longer and data controllers are better provisioned for data requests.

## Conclusion

With increasing interest in using non-health administrative data for research it is important to note the challenges that any such project might face. We hope that the narrative presented above, detailing the journey of a project through various stages of the necessary processes in Scotland, helps to highlight these challenges. Big data approaches are powerful, and have the potential to be faster, capture larger more representative samples, and collect more varied types of data than other research methods, such as survey studies. However, it is important for those considering pursuing non-health administrative data to appreciate the time and effort required to eventually acquire data, if at all. Again, these challenges largely arise from the infancy of non-health administrative data organisations and their processes, relative to their counterparts in health data research. While still prone to data access delays, the development of health administrative data research should be somewhat of a model, with clearer processes and more investment in capacity making it easier to produce important and impactful research using big data. Large-scale investment in administrative data research, similar to recent investments in health data research (e.g., Health Data Research UK) ^18^, is only possible if the situation becomes more conducive to research. As these investments are starting to be made, such as the UK and Scottish governments’ work in the Administrative Data Research Partnership, it is important to learn from previous efforts to make efficient use of resources. It is also important to incentivise organisations to participate fully in data sharing, and to encourage partnerships between data controllers and researchers. This may be achieved by the provision of funding and resources by research councils, by changing internal organisational goals, and by helping data controllers to benefit from research output ^19^. As has been noted with health administrative data, there is a significant potential for harm should non-health administrative data not be shared and used ^20^. As it stands, current attempts to obtain non-health administrative data are marked by uncertainty. Researchers are faced with a long journey through a complex and changeable landscape of permissions, approvals, and negotiations before reaching the prize of non-health administrative data. And non-health administrative data is quite the prize; with it, researchers have the potential to tackle the largest and most difficult problems faced by society.

## Summary

What was already known on the topic:

- Following the success of research using routinely-collect health administrative data, there is increasing availability of routinely-collected non-health administrative data for research purposes.
- The process of acquiring non-health administrative data is less well-established than that for health administrative data, and is constantly changing.

What this study adds:

- A comprehensive and factual narrative detailing the steps required to gain access to linked health and non-health administrative data, from the perspective of a researcher.
- A review of the problems and barriers facing linked data research, as well as a discussion of potential solutions.

## Data availability

No data are associated with this article.
