# Supplementary material for: Navigating the landscape of non-health administrative data in Scotland: A researcher’s narrative
Source: Wellcome Open Res. 2019 Oct 21;4:97. Originally published 2019 Jun 17. [Version 2] doi: 10.12688/wellcomeopenres.15336.2 (PMC6886011; doi:10.12688/wellcomeopenres.15336.2)
Supplement: Peer review history

# Peer review: Reviewer response for version 2

**DOI:** 10.21956/wellcomeopenres.17004.r36824

**Published:** 2019-11-29

**Authors:** Michael Fleming

**Affiliations:** 1 Institute of Health and Wellbeing, University of Glasgow, Glasgow, UK

I think the latest version of the manuscript is much improved and I am now happy that the authors have made efforts to address all previous comments where possible. I think the article reads more clearly now as a result.

 It is now clear to the reader that these issues described relate to non-health data and, whilst similar issues certainly still exist when linking health data, the process of linking health data is more straightforward and quicker by comparison.

 I think the paper highlights some very important issues and nicely summarizes some of the common problems when linking non-health data, whether it be trust or simply capacity issues. This clearly has an impact on research and the authors are correct to highlight the particularly detrimental impact on researchers on short term contracts, particularly PhD students. The authors also nicely summarize their arguments to have a more overarching and centralized approval process in place similar to PBPP for health data. This would be extremely beneficial. I just have two typographical errors to flag up. 

1. Typo on page 4 - "the organisations involved, their role and they type...." they should be the.
2. Typo on page 8 - "Indeed streamlining the data process........to data privacy nor at should it....." at should be deleted.

I confirm that I have read this submission and believe that I have an appropriate level of expertise to confirm that it is of an acceptable scientific standard.

# Peer review: Reviewer response for version 2

**DOI:** 10.21956/wellcomeopenres.17004.r36823

**Published:** 2019-10-21

**Authors:** Chris Playford

**Affiliations:** 1 University of Exeter, Exeter, UK

I have no further comments to make. The revised article is much improved.

I confirm that I have read this submission and believe that I have an appropriate level of expertise to confirm that it is of an acceptable scientific standard.

# Peer review: Reviewer response for version 1

**DOI:** 10.21956/wellcomeopenres.16744.r36032

**Published:** 2019-08-28

**Authors:** Michael Fleming

**Affiliations:** 1 Institute of Health and Wellbeing, University of Glasgow, Glasgow, UK

This is a useful personal account of a researcher’s struggles to gain access to, and link together, routine administrative datasets in Scotland and is a useful reference for other researchers aiming to do the same. It highlights a real issue which needs addressing and it is one which I have personally encountered. However, I feel that the article could be more general and broader in scope, with more developed conclusions and more discussion of some of the counter arguments for having such strict data governance in place. I have several comments which I feel would improve this article.

1. Throughout the manuscript the authors describe health data as ‘routinely collected health data’ and non-health data as ‘administrative data’. I do not like this terminology because healthcare data is also administrative data and is described this way throughout the literature. Therefore, I think the authors should stick to describing healthcare data as ‘routine administrative healthcare data’ and non-health data as ‘routine administrative non-health data’. We can of course get health and non-health data which isn’t administrative or routinely collected but these are not the focus of this manuscript.
2. The authors compound the problem by stating on more than one occasion that ‘researchers have recently extended their sights to routinely collected administrative data’ or that ‘access to administrative data is a relatively recent development’. These statements are not correct because healthcare data is (correctly) commonly thought of as routinely collected administrative data and has been accessed for decades. Be specific and highlight that you are referring to non-health data. Also, some context here would be helpful for the non-health data. What do the authors mean by more recently? Within the last 20, 10, or 5 years? 
3. The researcher’s perspective and narrative detailing the journey is very detailed and could, I feel, be greatly reduced whilst still getting the same message across. At present I feel this is too lengthy and dominates over the discussion of the wider issues and contexts which are covered in comparatively less in depth, but which are much more pertinent to the wider research community.
4. Following on from the last point, I feel that the title of the manuscript should therefore be reworded as it is a little misleading. The manuscript currently describes a particular set of circumstances arising from a research project aiming to analyse a particular set of administrative data sets so this should be specified and reflected more in the title. Alternatively, if the title remains the same then I think more focus needs to be put on the wider, more general, issues and less on the detailed specifics of the researchers own project narrative. I expected a more general review paper when I saw the title.
5. I agree with reviewer 1 that the authors need to give some more detail around SLS and highlight that this is a longstanding data resource which has been linked many times in the past hence accounting for the quicker access.
6. I agree with reviewer 1 that the authors should highlight that these difficulties have an important and detrimental knock-on effect particularly for PhD students who can’t gain access within the allotted study time, or indeed for researchers on short term contracts e.g. fellowships.
7. I think the authors need to provide more in the way of counter arguments. For example, why is governance around routine administrative data so strict and what are some of the drawbacks of making the approval processes easier? What are the authors own thoughts around the reasons for the current landscape being the way it is in terms of risks around accessing data? These processes are in place for a reason so it would be interesting to see some acknowledgement of these along with references to make this a more balanced account. Also, some more discussion around the background both in terms of the current processes for health (e.g. PBPP) and non-health data would be really useful to help navigate the reader and provide more general guidance around what the most important steps are when trying to apply to access health and non-health administrative datasets. I think the scope of the manuscript is too narrow and specific to the datasets within the researcher’s particular study and is not general enough given its very general and over-arching title.
8. These issues are still not just peculiar to non-health administrative data. Indeed, depending on the complexity of the project and the datasets involved, the approvals process to access administrative health data can still be much lengthier than it ought to be. I think this needs to be acknowledged within the manuscript. In terms of solutions for non-health data (and indeed health data), the authors mainly focus on adding capacity and improving large-scale investment. I’d like to have seen these ideas discussed in slightly more depth.

I confirm that I have read this submission and believe that I have an appropriate level of expertise to confirm that it is of an acceptable scientific standard, however I have significant reservations, as outlined above.

# Response

**Published:** 2019-10-18

**Authors:** Matthew Iveson

**Affiliations:** The University of Edinburgh, UK

Dear Dr Fleming, 

We very much appreciate the time and care that you have taken to review our manuscript. Your comments have helped to clarify several points and to develop the discussion of data access issues. We believe that the manuscript is more comprehensive and useful as a result of these changes. We have submitted an updated manuscript incorporating the comments of both reviewers. However, we would like to take the opportunity to outline our response to your individual comments below.

**Response to comments** 

This is a useful personal account of a researcher’s struggles to gain access to, and link together, routine administrative datasets in Scotland and is a useful reference for other researchers aiming to do the same. It highlights a real issue which needs addressing and it is one which I have personally encountered. However, I feel that the article could be more general and broader in scope, with more developed conclusions and more discussion of some of the counter arguments for having such strict data governance in place. I have several comments which I feel would improve this article.

**Response: We appreciate the reviewer’s concerns. From the start we have aimed this manuscript as an informative account of one specific set of projects, and we have acknowledged that this may not represent everyone’s experiences with routinely-collected data. Accurately portraying this journey necessitates a level of detail that we hope serves to motivate discussion about steps in the process which everyone undergoes. This being said, we agree with the reviewer and we have now attempted to broaden the discussion of the administrative data landscape and to reflect on changes to the system over time as well as organisational attitudes to data sharing (e.g., Process section, Page 11).**

**It is also not our intention to suggest that these processes should not be in place or that they should be less strict. Instead, we suggest that they are often disproportionate and fragile, being subject to changes and delays. We also do not suggest changing these processes necessarily, but instead advocate for better communication and collaboration between data controllers and researchers so as to manage expectations going in. As suggested by a later comment, we have added a paragraph to acknowledge that some complexity in the process is necessary to safeguard data (Process section, Page 11).**

Throughout the manuscript the authors describe health data as ‘routinely collected health data’ and non-health data as ‘administrative data’. I do not like this terminology because healthcare data is also administrative data and is described this way throughout the literature. Therefore, I think the authors should stick to describing healthcare data as ‘routine administrative healthcare data’ and non-health data as ‘routine administrative non-health data’. We can of course get health and non-health data which isn’t administrative or routinely collected but these are not the focus of this manuscript.

The authors compound the problem by stating on more than one occasion that ‘researchers have recently extended their sights to routinely collected administrative data’ or that ‘access to administrative data is a relatively recent development’. These statements are not correct because healthcare data is (correctly) commonly thought of as routinely collected administrative data and has been accessed for decades. Be specific and highlight that you are referring to non-health data. Also, some context here would be helpful for the non-health data. What do the authors mean by more recently? Within the last 20, 10, or 5 years?

**Response: We have amended our terms as the reviewer suggests. We made our original distinctions due to the focus of organisations such as the Administrative Data Research Network on obtaining non-health routinely-collected data. However, we appreciate that this may have caused some confusion. We have also clarified that much of the development has been regarding non-health administrative data. We have also replaced “recently” with “In the last decade” (Page 3).**

The researcher’s perspective and narrative detailing the journey is very detailed and could, I feel, be greatly reduced whilst still getting the same message across. At present I feel this is too lengthy and dominates over the discussion of the wider issues and contexts which are covered in comparatively less in depth, but which are much more pertinent to the wider research community.

**Response: The manuscript is intended as an account of a researcher’s journey to accessing administrative data. Its primary aim is to inform the reader – particularly new researchers – about the process and challenges, and therefore the level of detail is necessary. Whereas we do discuss the implications and the relevance to the wider data linkage scene, we agree that this was not sufficiently deep. However, the issues of ethics and governance have been discussed in greater detail elsewhere, and we have made sure to point the reader to them as well as raising the issues (e.g., Playford *et al*., 2016; Elias, 2018). As suggested, we have developed our discussion of the timing, process and capacity issues while referencing these other papers, while acknowledging that these are not new problems (Pages 10-12).**

Following on from the last point, I feel that the title of the manuscript should therefore be reworded as it is a little misleading. The manuscript currently describes a particular set of circumstances arising from a research project aiming to analyse a particular set of administrative data sets so this should be specified and reflected more in the title. Alternatively, if the title remains the same then I think more focus needs to be put on the wider, more general, issues and less on the detailed specifics of the researchers own project narrative. I expected a more general review paper when I saw the title.

**Response: We have amended the title to clarify that the manuscript is predominantly a narrative from a researcher’s perspective and relates primarily to non-health administrative data.**

I agree with reviewer 1 that the authors need to give some more detail around SLS and highlight that this is a longstanding data resource which has been linked many times in the past hence accounting for the quicker access.

**Response: As suggested by both reviewers, we have added an explanation of the SLS as a standing pre-linked resource where it is first introduced (Page 4). We have also added that SLS approvals and extraction are typically faster than non-SLS projects, referring to Figure 1.**

I agree with reviewer 1 that the authors should highlight that these difficulties have an important and detrimental knock-on effect particularly for PhD students who can’t gain access within the allotted study time, or indeed for researchers on short term contracts e.g. fellowships.

**Response: We have highlighted the particular issue for PhD and early-career researchers in the second paragraph on Timing (page 10).**

I think the authors need to provide more in the way of counter arguments. For example, why is governance around routine administrative data so strict and what are some of the drawbacks of making the approval processes easier? What are the authors own thoughts around the reasons for the current landscape being the way it is in terms of risks around accessing data? These processes are in place for a reason so it would be interesting to see some acknowledgement of these along with references to make this a more balanced account. Also, some more discussion around the background both in terms of the current processes for health (e.g. PBPP) and non-health data would be really useful to help navigate the reader and provide more general guidance around what the most important steps are when trying to apply to access health and non-health administrative datasets. I think the scope of the manuscript is too narrow and specific to the datasets within the researcher’s particular study and is not general enough given its very general and over-arching title.

**Response: We would like to thank the reviewer for highlighting the need for a balanced discussion regarding the information governance process. We agree that the process is complex for a reason and have incorporated these suggestions into the manuscript. In particular, we have added the following to the Process section (Page 11):**

- “It is worth noting that some degree of complexity is necessary given the sensitivity and scale of the data being requested and the resulting risk to privacy and impact of improper use. The variety of data access procedures described here are in place to protect the privacy of individuals and their data, and it is important that researchers demonstrate their plans for and commitment to minimise any risks. Furthermore data controllers such as NRS have responsibilities to care for the data under their charge which outweigh their responsibilities to share data for research. We therefore do not suggest that the data access process should be less thorough or strict. Indeed, streamlining the data access process (e.g., uniting all non-health data access approvals) should not come at the cost of an increased risk to data privacy nor at should it damage data controllers’ responsibilities or reputation. However, improvements can be made to make the journey to obtaining data clearer and smoother.”

**Notably, we do not suggest that the governance and access process should become less strict or easier. Instead, we suggest that the process could be smoother and more streamlined, and that timescales could be reduced. This requires further investment in capacity and better communication between researchers and data controllers. A deeper discussion of the governance process is provided by other papers (e.g., Playford *et al*., 2016; Elias, 2018), and we have noted this throughout the Issues section (Pages 10-12).**

**More detail about the current processes is included in the sections about Stage 1-5. For example, the background to the PBPP process is described in Stage 3 and in the Process section. We have further clarified and distinguished the steps needed to access health and non-health data in the Process section (Page 11).**

These issues are still not just peculiar to non-health administrative data. Indeed, depending on the complexity of the project and the datasets involved, the approvals process to access administrative health data can still be much lengthier than it ought to be. I think this needs to be acknowledged within the manuscript. In terms of solutions for non-health data (and indeed health data), the authors mainly focus on adding capacity and improving large-scale investment. I’d like to have seen these ideas discussed in slightly more depth.

**Response: The issues of complexity and timing have been raised in regards to health-related administrative data elsewhere. For example, an early attempt to link the same cohort to routinely-collected health data across the UK found that the process (pre-PBPP) took almost 2 years and required some 210 documents (Brett & Deary, 2014). Since this paper the process for accessing health data has been streamlined (e.g., the introduction of the PBPP process) and has gotten faster for the majority of users. We have highlighted the development in health data access (and the previous paper) in the Process section (Pages 11-12), in particular noting that access to health data has become streamlined if not faster. We have also acknowledged that this streamlining is not perfect, and that health data access can still be longer than is necessary in both the Timing section (Page 10) and the Process section (Page 12).**

**We have discussed the potential solutions to data access problems – capacity, investment and communication – in more detail in the relevant sections and in the Conclusion (Page 13).**

# Peer review: Reviewer response for version 1

**DOI:** 10.21956/wellcomeopenres.16744.r35782

**Published:** 2019-06-21

**Authors:** Chris Playford

**Affiliations:** 1 University of Exeter, Exeter, UK

This is a helpful article that serves to illustrate the current challenges for researchers working with administrative data in Scotland. I recommend this article be approved for indexing because the account provides a useful contribution to understanding the current governance of administrative data access and the impact this can have on academic research. I have a number of recommendations for how the article might be improved. The first are minor points about clarifying the existing text. The final part of this review provides suggestion of how the scope of the conclusions might be developed further for the benefit of researchers, funders, and those working with administrative data in the UK. This is important for improving access to administrative data for future researchers and recognising the broader issues in which this process is currently contextualised. This final set of recommendations help clarify my response to the question: *“Does the article adequately reference differing views and opinions?”*

 Firstly, here are my specific points of clarification:

 On page 3, you mentioned the Scottish Longitudinal Study (SLS). It would be helpful to provide more context for readers unfamiliar with the SLS (see https://sls.lscs.ac.uk/about/). For instance, as you are aware, the SLS is a “standing resource” whereas the other linked data require that the data are linked for the first time. You mention this on page 6 but it would be useful to know earlier in the article. This explains in part why the data extraction and indexing differ so greatly, as you show helpfully in Figure 1.

 Table 1 has a number of blank cells where I would have expected numeric figures to be shown – this needs rectifying.

 Please be consistent when describing dates – I would recommend you always include the year otherwise it is easy to lose track of the general timescale. For example, on page 6 you report a number of dates without the year listed.

 Box 1 on page 5 is helpful as a means of comparing the process when using the SLS data. It is helpful to reiterate to the reader frequently that the SLS is quite different to the other administrative data projects which have not been previously linked.

 On page 7, you refer to the funding timescale of the ADRC-S and ADRN. I would suggest in your conclusion that you add that that these were preceded in Scotland by the Administrative Data Liaison Service (ADLS) and have been superseded by the Administrative Data Research Partnership (ADRP). It is useful for the reader to be aware of this evolution. This is relevant so that those working in the field can see the need to learn from previous initiatives. As a broader point, a further challenge to researchers in Scotland and the UK is that much of the documented activity and resources these projects created does not appear to be available as much of the web content has either been removed. This is an impediment to current and future researchers seeking to learn about potential administrative datasets, processes of accessing data and the reproducibility of administrative data research (see Playford *et al*., (2016) ^1^).

 Here are some webpages that may be helpful.

 Administrative Data Liaison Service (ADLS)

https://www.spi.ox.ac.uk/administrative-data-liaison-service-2014

https://www.bristol.ac.uk/media-library/sites/cmpo/migrated/documents/elliot.pdf

 Administrative Data Research Network (ADRN)

https://esrc.ukri.org/research/our-research/administrative-data-research-network/

 For a general overview of the ADRN and lessons learned, see Elias (2018) ^2^.

 New Administrative Data Research Partnership (ADRP)

https://esrc.ukri.org/news-events-and-publications/news/news-items/addressing-major-societal-challenges-by-harnessing-government-data/

 Scottish ADRP

http://www.epcc.ed.ac.uk/blog/2018/scottish-administrative-data-research-partnership

 On page 7, when you describe the issues faced, I would suggest you add that the timescales involved in this project would have precluded a PhD student from using linked administrative data as part of their thesis.

 You make a number of important points when reflecting on the number of forms that were requested and the changes to the process that were developed during the project.

 My final points relate to developing your conclusions further. These reflect briefly on the implications for researchers wishing to use administrative data. It would be helpful for the reader to understand the administrative data context in Scotland better. The following points include some references you may find useful.

 Initiatives such as the recent Administrative Data Research Partnership (ADRP) indicate the substantial investment by the UK government into the use of administrative data for research purposes in the social sciences. There is a clear desire to use this money wisely and efficiently. To do so, I would suggest that we must learn from previous efforts (see also Elias (2018) ^2^). Your paper is a practical exemplar of the challenges faced by researchers working in the field. It is more broadly recognised that accessing administrative data is currently tricky and time-consuming (Connelly *et al*., (2016) ^3^, Harron *et al*., (2017) ^4^).

 I would encourage you to reflect in your conclusions on the wider organisational context in which administrative data research occurs. For example, although initially focusing on the legal gateways through which data could be accessed, Laurie and Stevens (2016) ^5^ identified that problematic organizational culture (particularly the perception of risk) was a significant barrier to proportionate governance. Sexton *et al*. (2017, p.327) ^6^ argue that: *“In trustworthy systems and processes, a balance must be struck between appropriate monitoring in the system whilst ensuring against excessive auditing that may counterproductively contribute to the erosion of trust.”* Whilst describing administrative data access in the USA, Card *et al*. (2010) ^7^ provide some helpful suggestions of how to incentivise administrative data access and output for agencies involved. I would encourage you to reflect further on these points in your conclusions. Finally, there is a risk of potential harm due to the non-use of data (Jones *et al*., (2017) ^8^). It is therefore important that the barriers that you describe are overcome if others are to benefit from this work.

I confirm that I have read this submission and believe that I have an appropriate level of expertise to confirm that it is of an acceptable scientific standard.

## References

[1] : Administrative social science data: The challenge of reproducible research. *Big Data & Society*.2016;3(2) : 10.1177/2053951716684143 10.1177/2053951716684143

[2] : The UK Administrative Data Research Network: Its Genesis, Progress, and Future. *The ANNALS of the American Academy of Political and Social Science*.2018;675(1) : 10.1177/0002716217741279 184-201 10.1177/0002716217741279

[3] : The role of administrative data in the big data revolution in social science research. *Soc Sci Res*.59: 10.1016/j.ssresearch.2016.04.015 1-12 10.1016/j.ssresearch.2016.04.015 27480367

[4] : Challenges in administrative data linkage for research. *Big Data Soc*.2017;4(2) : 10.1177/2053951717745678 2053951717745678 10.1177/2053951717745678 30381794PMC6187070

[5] : Developing a Public Interest Mandate for the Governance and Use of Administrative Data in the United Kingdom. *Journal of Law and Society*.2016;43(3) : 10.1111/j.1467-6478.2016.00759.x 360-392 10.1111/j.1467-6478.2016.00759.x

[6] : A balance of trust in the use of government administrative data. *Archival Science*.2017;17(4) : 10.1007/s10502-017-9281-4 305-330 10.1007/s10502-017-9281-4

[7] : Expanding Access to Administrative Data for Research in the United States. *SSRN Electronic Journal*.2010; 10.2139/ssrn.1888586 10.2139/ssrn.1888586

[8] : The other side of the coin: Harm due to the non-use of health-related data. *Int J Med Inform*.97: 10.1016/j.ijmedinf.2016.09.010 43-51 10.1016/j.ijmedinf.2016.09.010 27919394

# Response

**Published:** 2019-10-18

**Authors:** Matthew Iveson

**Affiliations:** The University of Edinburgh, UK

Dear Dr Playford, 

We very much appreciate the time and care that you have taken to review our manuscript. Your comments have helped to clarify several points and to develop the discussion of data access issues. We believe that the manuscript is more comprehensive and useful as a result of these changes. We have submitted an updated manuscript incorporating the comments of both reviewers. However, we would like to take the opportunity to outline our response to your individual comments below.

**Response to comments** 

This is a helpful article that serves to illustrate the current challenges for researchers working with administrative data in Scotland. I recommend this article be approved for indexing because the account provides a useful contribution to understanding the current governance of administrative data access and the impact this can have on academic research. I have a number of recommendations for how the article might be improved. The first are minor points about clarifying the existing text. The final part of this review provides suggestion of how the scope of the conclusions might be developed further for the benefit of researchers, funders, and those working with administrative data in the UK. This is important for improving access to administrative data for future researchers and recognising the broader issues in which this process is currently contextualised. This final set of recommendations help clarify my response to the question: “Does the article adequately reference differing views and opinions?”

Firstly, here are my specific points of clarification:

- On page 3, you mentioned the Scottish Longitudinal Study (SLS). It would be helpful to provide more context for readers unfamiliar with the SLS (see https://sls.lscs.ac.uk/about/). For instance, as you are aware, the SLS is a “standing resource” whereas the other linked data require that the data are linked for the first time. You mention this on page 6 but it would be useful to know earlier in the article. This explains in part why the data extraction and indexing differ so greatly, as you show helpfully in Figure 1.

**Response: As suggested, we have added explanation of the SLS as a standing pre-linked resource where it is first introduced (Page 4). We have also added that SLS approvals and extraction are typically faster than non-SLS projects, referring to Figure 1. **

- Table 1 has a number of blank cells where I would have expected numeric figures to be shown – this needs rectifying.

**Response: The blank columns indicated where an organisation did not have a ‘data controller’ row, and therefore did not have an applicable data type. We have amended Table 1 to remove the ‘Type of data’ column and instead expand the ‘Role’ column to reference the type of data for a given data controller.**

- Please be consistent when describing dates – I would recommend you always include the year otherwise it is easy to lose track of the general timescale. For example, on page 6 you report a number of dates without the year listed.

**Response: We have added the year to all dates, as suggested.**

- Box 1 on page 5 is helpful as a means of comparing the process when using the SLS data. It is helpful to reiterate to the reader frequently that the SLS is quite different to the other administrative data projects which have not been previously linked.

**Response: We have strengthened the description of the SLS in Box 1 to highlight the differences in procedure/requirements between the SLS and the two ‘unlinked’ census projects. We have also added sentences to Stage 3 (page 6) and Stage 4 (page 7) to better highlight that the SLS process is markedly different for these stages in particular.**

- On page 7, you refer to the funding timescale of the ADRC-S and ADRN. I would suggest in your conclusion that you add that that these were preceded in Scotland by the Administrative Data Liaison Service (ADLS) and have been superseded by the Administrative Data Research Partnership (ADRP). It is useful for the reader to be aware of this evolution. This is relevant so that those working in the field can see the need to learn from previous initiatives. As a broader point, a further challenge to researchers in Scotland and the UK is that much of the documented activity and resources these projects created does not appear to be available as much of the web content has either been removed. This is an impediment to current and future researchers seeking to learn about potential administrative datasets, processes of accessing data and the reproducibility of administrative data research (see Playford et al., (2016) ^1^).

Here are some webpages that may be helpful.

Administrative Data Liaison Service (ADLS)

https://www.spi.ox.ac.uk/administrative-data-liaison-service-2014

https://www.bristol.ac.uk/media-library/sites/cmpo/migrated/documents/elliot.pdf

Administrative Data Research Network (ADRN)

https://esrc.ukri.org/research/our-research/administrative-data-research-network/

For a general overview of the ADRN and lessons learned, see Elias (2018) ^2^.

New Administrative Data Research Partnership (ADRP)

https://esrc.ukri.org/news-events-and-publications/news/news-items/addressing-major-societal-challenges-by-harnessing-government-data/

Scottish ADRP

http://www.epcc.ed.ac.uk/blog/2018/scottish-administrative-data-research-partnership

**Response: We would like to thank the reviewer for their suggestions and for the links to the relevant organisations. We have added detail of the preceding and succeeding organisations to the Timing section where we reference the funding timescale of the ADRC-S (page 10) and have highlighted the potential loss of important documentation. We have also referenced Elias (2018) and Playford *et al*. (2016) in our attempts to better contextualise the ADRC-S and its evolution.**

On page 7, when you describe the issues faced, I would suggest you add that the timescales involved in this project would have precluded a PhD student from using linked administrative data as part of their thesis.

**Response: We have noted this in the second paragraph on Timing (page 10).**

You make a number of important points when reflecting on the number of forms that were requested and the changes to the process that were developed during the project.

**Response: We thank the reviewer for the comment, and hope that presenting simple statistics such as the number of forms required helps researchers to manage their expectations going into administrative data research.**

My final points relate to developing your conclusions further. These reflect briefly on the implications for researchers wishing to use administrative data. It would be helpful for the reader to understand the administrative data context in Scotland better. The following points include some references you may find useful.

Initiatives such as the recent Administrative Data Research Partnership (ADRP) indicate the substantial investment by the UK government into the use of administrative data for research purposes in the social sciences. There is a clear desire to use this money wisely and efficiently. To do so, I would suggest that we must learn from previous efforts (see also Elias (2018) ^2^). Your paper is a practical exemplar of the challenges faced by researchers working in the field. It is more broadly recognised that accessing administrative data is currently tricky and time-consuming (Connelly et al., (2016) ^3^, Harron et al., (2017) ^4^).

**Response: We have added reference to the significant investment by the UK government, the drive for efficiency, and the need to learn lessons to the Conclusion (Page 13). We have also referenced this issue in the Capacity section (Pages 12-13), as any investment needs to be sufficiently targeted for efficiency.**

I would encourage you to reflect in your conclusions on the wider organisational context in which administrative data research occurs. For example, although initially focusing on the legal gateways through which data could be accessed, Laurie and Stevens (2016) ^5^ identified that problematic organizational culture (particularly the perception of risk) was a significant barrier to proportionate governance. Sexton et al. (2017, p.327) ^6^ argue that: “In trustworthy systems and processes, a balance must be struck between appropriate monitoring in the system whilst ensuring against excessive auditing that may counterproductively contribute to the erosion of trust.” Whilst describing administrative data access in the USA, Card et al. (2010) ^7^ provide some helpful suggestions of how to incentivise administrative data access and output for agencies involved. I would encourage you to reflect further on these points in your conclusions. Finally, there is a risk of potential harm due to the non-use of data (Jones et al., (2017) ^8^). It is therefore important that the barriers that you describe are overcome if others are to benefit from this work.

**Response: We have added discussion of the organisational context, including the need for a balanced governance procedure, in the Process section (Page 11). We have also added a note regarding incentives and the consequences of data non-use in the Conclusion (Page 13). We would also like to thank the reviewer for the suggested references, and we have included them in the relevant sections.**
